# Changes in Buprenorphine-Naloxone and Opioid Pain Reliever Prescriptions After the Affordable Care Act Medicaid Expansion

**DOI:** 10.1001/jamanetworkopen.2018.1588

**Published:** 2018-08-17

**Authors:** Brendan Saloner, Jonathan Levin, Hsien-Yen Chang, Christopher Jones, G. Caleb Alexander

**Affiliations:** 1 Department of Health Policy & Management, Johns Hopkins Bloomberg School of Public Health, Baltimore, Maryland; 2 Center for Drug Safety and Effectiveness, Johns Hopkins Bloomberg School of Public Health, Baltimore, Maryland; 3 National Mental Health and Substance Use Policy Laboratory, Substance Abuse and Mental Health Services Administration, US Department of Health and Human Services, Washington, DC; 4 Department of Epidemiology, Johns Hopkins Bloomberg School of Public Health, Baltimore, Maryland; 5 Division of General Internal Medicine, Johns Hopkins Medicine, Baltimore, Maryland

## Abstract

**Importance:**

Expanding Medicaid eligibility could affect prescriptions of buprenorphine with naloxone, an established treatment for opioid use disorder, and opioid pain relievers (OPRs).

**Objective:**

To examine changes in prescriptions of buprenorphine with naloxone and OPRs after the US Affordable Care Act Medicaid expansion.

**Design, Setting, and Participants:**

In this cohort study, longitudinal, patient-level, retail pharmacy claims were extracted from IQVIA real-world data from an anonymized, longitudinal, prescription database. The sample included 11.9 million individuals who filled 2 or more prescriptions for a prescription opioid during at least 1 year between January 1, 2010, and December 31, 2015, from California, Maryland, and Washington (expansion states) and Florida and Georgia (nonexpansion states). Data analysis was conducted from August 1, 2017, to May 31, 2018. Data were aggregated to county-year observations (N = 2082) and linked to county-level covariates. For each outcome, a difference-in-differences regression model was estimated comparing changes before and after expansion in expansion vs nonexpansion counties. Models were adjusted for county demographics, uninsured rate, and overdose mortality in the baseline year (2010).

**Exposures:**

Presence of Medicaid expansion in the year.

**Main Outcomes and Measures:**

For buprenorphine with naloxone and OPRs, rates per 100 000 county residents were calculated separately for any prescriptions overall and by different payment sources. Mean days of medication per county among people filling prescriptions for these agents were also determined.

**Results:**

The study sample included 11.9 million individuals (expansion states: 40.9% men; mean [SD] age, 44.1 [13.8] years; nonexpansion states: 41.0% men; mean [SD] age, 43.7 [13.7] years). In expansion counties, 68.8 individuals per 100 000 county residents filled buprenorphine with naloxone and 5298.3 filled OPR prescriptions in 2010. After expansion, buprenorphine with naloxone fills per 100 000 county residents increased significantly in expansion relative to nonexpansion counties (8.7; 95% CI, 1.7 to 15.7). Opioid pain reliever fills per 100 000 county residents did not significantly change in expansion counties relative to nonexpansion counties (327.4; 95% CI −202.5 to 857.4). The rate of OPRs per 100 000 county residents paid for by Medicaid significantly increased (374.0; 95% CI, 258.3 to 489.7). There were no significant changes in days per 100 000 county residents of either medication after expansion.

**Conclusions and Relevance:**

Medicaid expansion significantly increased buprenorphine with naloxone prescriptions per 100 000 county residents in expansion counties, suggesting that expansion improved access to opioid use disorder treatment. Expansion did not significantly increase the overall rate per 100 000 county residents of OPR prescriptions, but increased the population with OPRs paid for by Medicaid. This finding therefore suggests the growing importance of Medicaid in pain management and addiction prevention.

## Key Points

**Question:**

Did Medicaid expansion under the US Affordable Care Act change prescription fills for buprenorphine with naloxone, a treatment for opioid use disorder, and opioid pain relievers?

**Findings:**

In this cohort study using difference-in-differences analysis of all-payer prescription fill data from 5 states, Medicaid expansion was associated with a significant overall increase in people filling prescriptions for buprenorphine with naloxone. Expansion was not associated with changes in fills per 100 000 county residents of opioid pain relievers overall, but significantly more people filled prescriptions for opioid pain relievers paid for specifically by Medicaid.

**Meaning:**

Medicaid expansion may increase the role of states in providing opioid use disorder treatment and in paying for opioid pain relievers for pain management.

## Teaser

This pharmacoepidemiology study compares changes in prescriptions for opioid pain relievers and for buprenorphine with naloxone in 5 states that did vs did not expand Medicaid under the Affordable Care Act (ACA) in 2014.

## Introduction

Between 2010 and 2016, the uninsured rate in the United States declined from 16% to 9%, largely because of provisions of the Affordable Care Act (ACA).^1^ The main coverage provisions of the ACA were implemented in 2014, including Medicaid expansion to individuals below 138% of the federal poverty level and health insurance exchanges with sliding-scale subsidies for individuals above 100% of the poverty level. Although the exchanges are national, Medicaid expansion is an optional program that was initially adopted by the District of Columbia and 25 states, with several additional states expanding such coverage by 2018. Coverage gains have been larger in Medicaid-expansion states than in nonexpansion states.^1^

The increases in insurance coverage under the ACA have occurred alongside increasing injuries and deaths attributable to opioids. Opioids, including both prescription opioid pain relievers (OPRs) and heroin and illicit fentanyl, now account for almost two-thirds of all drug overdose deaths.^2^ Overdoses have quadrupled since the late 1990s and are now the leading cause of injury death in the United States.^2^

Concerns have been raised by ACA opponents that the Medicaid expansion has made the opioid crisis worse, contending that new enrollees could use their insurance to gain access to low-cost OPRs, increasing rates of abuse and diversion.^3^ Fatal drug overdoses increased more rapidly in Medicaid-expansion states than nonexpansion states from 2013 to 2015, but this trend was higher in years prior to Medicaid expansion and thus unlikely to be caused by the ACA.^4^ A recent analysis found that opioid-related hospitalizations did not increase more rapidly in expansion vs nonexpansion states.^5^ Even if fatal overdoses and hospitalizations were not affected by the ACA, it is still possible that Medicaid expansion could have increased OPR prescribing. One recent study compared trends in the volume of OPRs paid for by Medicaid in expansion and nonexpansion states and found that Medicaid-reimbursed OPRs increased in both states, but did not significantly differ by Medicaid-expansion status. This study, however, did not examine corresponding changes in other sources of payment, leaving open the question of whether there were offsetting changes in out-of-pocket payment or private insurance.^6^

Conversely, the ACA insurance expansion could play a role in combating the opioid epidemic by increasing access to treatment of opioid use disorder, which is a required benefit in the ACA insurance exchanges and Medicaid-expansion plans.^7^ Opioid use disorder can be managed beneficially with medication, yet most individuals with opioid use disorder do not receive any treatment.^8^ Buprenorphine, a partial opioid agonist medication that can be prescribed by clinicians who possess a federal waiver, is likely to be one treatment that is especially sensitive to health insurance coverage changes. Although some buprenorphine formulations are indicated for pain relief (eg, the transdermal patch), combined buprenorphine and naloxone is the most common formulation and is approved by the US Food and Drug Administration for treatment of opioid dependence (and rarely used for pain management).^9^ Consultation with an office-based prescriber is difficult for uninsured patients to access compared with patients with Medicaid or private insurance, and buprenorphine with naloxone is an expensive medication for patients paying out of pocket.^10^ The total volume of buprenorphine with naloxone reimbursed by Medicaid increased more in expansion states compared with nonexpansion states,^6,11,12^ but as with OPRs, research has not examined how much of this increase is accounted for by new patients initiating treatment vs patients shifting from other payers to Medicaid after expansion.

We quantified changes in OPR and buprenorphine with naloxone prescription fills after the ACA using longitudinal, patient-level, retail pharmacy claims data from an all-payer database in 5 states. We hypothesized that the number of patients filling prescriptions for both OPRs and buprenorphine with naloxone would increase after Medicaid expansion in expansion states relative to nonexpansion states. We also hypothesized that the share of both OPRs and buprenorphine with naloxone reimbursed by Medicaid would increase after expansion and that cash payment would decrease because of increased insurance coverage.

## Methods

### Setting and Selection of Study Participants

We used the IQVIA real-world anonymized, longitudinal, prescription data on individuals prescribed medications in California, Florida, Georgia, Maryland, and Washington between January 1, 2010, and December 31, 2015. During the time of our study, the database captured approximately 75% to 80% of retail transactions in the United States that are automatically reported to IQVIA through weekly feeds from retail, food store, independent, and mass merchandiser pharmacies. These anonymized, all-payer claims data contain detailed information for each prescription, including the fill date, days’ supply, and payment type. These data have been used previously to study buprenorphine and OPR fills associated with policy.^13,14^ Analysis was conducted from August 1, 2017, to May 31, 2018. Analysis of secondary, deidentified data is considered exempt by the Johns Hopkins Institutional Review Board. This study followed the Strengthening the Reporting of Observational Studies in Epidemiology (STROBE) reporting guideline.

The extracted data obtained for the study included the full set of prescription records for individuals filling 2 or more prescriptions for any opioid (either OPRs or buprenorphine with naloxone) during the study period with at least 1 claim from the 5 study states. The requirement of 2 opioid claims was made to identify people who might be more at risk of chronic opioid use. Using National Drug Codes, we separately identified schedule II to IV nonbuprenorphine OPRs and buprenorphine with naloxone.^15^ Methadone in retail pharmacy claims is included as an OPR because it can be prescribed only for pain management (ie, not opioid use disorder). We restricted our sample to adults aged 18 to 65 years. We assigned people to the county where they retrieved the majority of their prescriptions. Our final sample included 162.9 million transactions for 11.9 million individuals. The individual records were then aggregated to county-year summary observations, which was our main unit of analysis. Across the 5 states there were 347 counties that were tracked through the 6 study years (2082 county-years). Of these, there was at least 1 individual with prescriptions in 2000 county-years, and we imputed a 0 value for 82 county-years during which there were no records of filled prescriptions.

California, Maryland, and Washington expanded Medicaid under the ACA statewide January, 1 2014, and Florida and Georgia did not expand during the study period. In addition, California obtained a federal waiver that allowed counties to adopt Medicaid expansion beginning in 2011. We thus defined Medicaid expansion as present in counties that had an expansion for the full calendar year using expansion dates reported in a prior study.^16^ There were 121 counties in the Medicaid-expansion states and 333 county-years in which Medicaid expansion was present.

### Prescription-Related Outcomes

Using the prescription data, we calculated the number of people in our sample during each county-year who filled a prescription for an OPR or buprenorphine with naloxone. These counts provided a basis for examining trends over time, but by design, our data excluded some individuals, such as those with only 1 prescription for an opioid, people receiving medication from nonretail sources, and people younger than 18 years or older than 65 years. Therefore, our data should not be interpreted as measuring the county-wide prevalence of either medication during a given year.

We calculated fill rates of OPR or buprenorphine with naloxone per 100 000 population across all payers using annual counts for each medication and annual county-level population estimates from the American Community Survey.^17^ We also quantified prescription rates per 100 000 county residents for OPR and buprenorphine with naloxone in counties separately paid for by the 4 main sources of payment: Medicaid (including both fee-for-service and managed care), cash, private insurance, and Medicare. Because the same individual could fill prescriptions with more than 1 payer, the sum of all rates per 100 000 county residents by payer equals more than the overall mean county fill rate per 100 000 county residents. To examine changes in duration of medication filled, we also measured the mean number of days of filled medication in each county-year among people who had at least 1 prescription filled.

### County Covariates

We included county-level measures from the Area Health Resource File captured during 2010, our baseline study year, including the physician-to-population ratio, the uninsured rate, median income, percentage of women, and percentage from major racial/ethnic groups, including non-Hispanic white, non-Hispanic black, Hispanic, and other non-Hispanic.^18^ We also included the drug overdose death rate in the county during 2010 using county-level categories created by the National Center for Health Statistics.^19^

### Statistical Analysis

We first summarized trends associated with prescription rates overall and by source of payment, and annual duration of treatment for OPRs and buprenorphine with naloxone during the study period. We stratified the sample by Medicaid-expansion vs nonexpansion counties and created annual plots showing the rate of individuals filling prescriptions for OPRs and buprenorphine with naloxone, and the same rates by source of payment. In all analyses, we weighted each observation by the county’s population using data from the American Community Survey.

Next, we estimated a difference-in-differences regression model that identified the outcomes of Medicaid expansion by comparing the pre- and post-Medicaid-expansion change in expansion counties with the changes in nonexpansion counties during the same period. By focusing on temporal changes between the 2 groups, difference-in-differences models minimize bias that might otherwise arise from cross-sectional comparisons because of selection into Medicaid expansion. The models assume that nonexpansion states provide a counterfactual trend for counties in expansion states. This assumption is more plausible if the trend in outcomes between counties in expansion and nonexpansion states did not differ significantly in years prior to the expansion, and thus we tested for parallel trends in pre-expansion years (eTable 1 in the Supplement).

Models included state and year fixed effects. All models were estimated using ordinary least-squares regression, which is suited for interpreting interactions.^20^ Standard errors were clustered at the state level using the clustered sandwich estimator.^21^ In unadjusted analysis, we calculated standardized effect sizes for unadjusted comparisons focusing on moderate or greater effect sizes (>0.5).^22^ We calculated 95% CIs for all regression estimates and adopted 2-tailed, unpaired *P* < .05 as the threshold for statistical significance. Data were analyzed using Stata, version 15.1 (StataCorp).

### Sensitivity Analyses

We considered several sensitivity analyses. First, we tested whether our results were different using an alternative sample that includes individuals who are consistently observed in the prescription fill data. This alternative (constant) sample consists of 4.7 million individuals who have records in all of the study years for any medications (not exclusively opioids). Second, as an alternative to the difference-in-differences approach, we examined a regression model that includes an additional interaction term with the county’s uninsured rate in 2010, an approach that has been used to test for within-state variation based on expected uptake of insurance.^23^ Third, we tested the sensitivity of our analyses to excluding data from each of the states by dropping each state 1 at a time and rerunning the models. Fourth, we reran our models applying inverse probability of treatment weights. These weights allowed us to adjust our sample of nonexpansion counties to more closely resemble our expansion counties. We generated our weights using the county-level covariates from the baseline year. In our regression analysis, we created a new combined weight by multiplying inverse probability of treatment weight by our population weight.^24^

## Results

Table 1 examines the mean age and sex of individuals in the IQVIA study sample and other county-level characteristics for their counties of residence from the American Community Survey in 2010. Sampled individuals in expansion counties included 40.9% men; mean (SD) age was 44.1 (13.8) years. Individuals in nonexpansion counties had similar demographics (41.0% men; mean [SD] age, 43.7 [13.7] years). Counties in the Medicaid-expansion states included more people of Hispanic and other race/ethnicity and fewer African American and non-Hispanic white individuals than nonexpansion states. The counties in expansion states had higher median incomes, lower uninsured rates, and more primary care physicians.

**Table 1.** Characteristics of Expansion and Nonexpansion Counties in 2010^a^

Characteristic	County Mean in 2010, %		Effect Size (95% CI)
	Expansion States	Nonexpansion States	
Demographics of individuals filling prescriptions			
Men	40.9	41.0	−0.006 (−0.007 to −0.005)
Age, mean (SD), y	44.1 (13.8)	43.7 (13.7)	0.003 (0.002 to 0.005)
Demographics of counties			
Adults (age >18 y)	63.9	62.2	−0.14 (−0.37 to 0.08)
Non-Hispanic white	45.9	56.9	−0.04 (−0.27 to 0.18)
Non-Hispanic black	8.2	20.4	1.23 (0.99 to 1.47)
Non-Hispanic other	13.9	3.5	−1.41 (−1.66 to −1.17)
Hispanic	31.0	18.1	−0.89 (−1.12 to −0.66)
Women	50.4	51.1	−0.006 (−0.23 to 0.21)
Rural	7.4	14.3	0.60 (0.38 to 0.83)
Uninsured	23.5	28.7	1.34 (1.1 to 1.58)
Median annual income (IQR), $	59 643 (51 017-68 596)	45 577 (40 258-48 973)	−1.14 (−1.38 to −0.90)
Primary care physician to population ratio	0.77	0.67	−0.69 (−0.91 to −0.46)
County overdose death rate			
<10 per 100 000 residents	29.5	32.1	0.39 (0.17 to 0.61)
10-20 per 100 000 residents	67.4	57.2	−0.28 (−0.51 to −0.06)
>20 per 100 000 residents	3.2	10.7	−0.18 (−0.40 to 0.04)
No. of counties	121	226	

Abbreviation: IQR, interquartile range.

^a^ Total of 347 counties. Demographics of individuals filling prescriptions calculated from the study sample derived from IQVIA data. Demographic characteristics derived from 2010 values from the Area Health Resource File and county overdose death rate derived from the Centers for Disease Control and Prevention National Center for Health Statistics Estimates. Counties were weighted by their population in 2010. Effect size differences were calculated using Cohen *d* statistic.

### Opioid Pain Relievers

**Figure 1.**
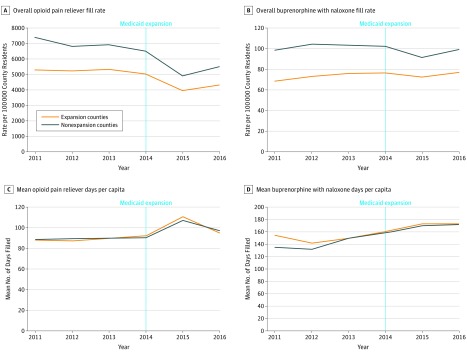
Trends in Overall Rate of People Filling Prescriptions and Days Filled for Opioid Pain Relievers and Buprenorphine With Naloxone in Medicaid Expansion and Nonexpansion Counties Analysis of IQVIA prescription claims data on overall opioid pain relievers (A), overall buprenorphine with naloxone (B), mean opioid pain reliever days per 100 000 county residents (C), and mean buprenorphine with naloxone days per 100 000 county residents (D) aggregated to county-years from California, Maryland, and Washington (Medicaid expansion counties) and Florida and Georgia (nonexpansion counties), N = 2082 county-years. County-years are weighted by the county population.

**Figure 2.**
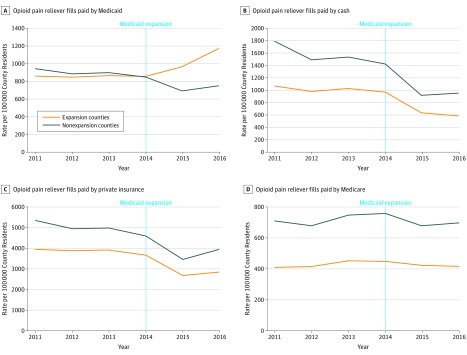
Trends in Rate of People Filling Prescriptions for Opioid Pain Relievers by Payer Analysis of IQVIA prescription claims data on prescriptions paid by Medicaid (A), cash (B), private insurance (C), and Medicare (D) aggregated to county-years from California, Maryland, and Washington (Medicaid expansion counties) and Florida and Georgia (nonexpansion counties), N = 2082 county-years. County-years are weighted by the county population.

For the 2010-2015 period, fills per 100 000 residents for OPRs declined from 5298.3 to 4316.2 in expansion counties and from 7404.2 to 5510.6 in nonexpansion counties (Figure 1). The mean days with filled OPRs increased for both groups of counties from 88.1 to 94.5 in expansion counties and from 88.4 to 96.5 in nonexpansion counties (Figure 1). The rate of individuals filling OPRs reimbursed by Medicaid per 100 000 increased in expansion counties from 859.5 to 1170.9, while declining in nonexpansion counties from 943.4 to 750.6 (Figure 2). The rate of individuals filling OPRs paid for by all other forms of payment (private insurance, cash, and Medicare) declined in both expansion and nonexpansion counties during the study period (Figure 2).

In difference-in-differences analysis, there was no statistically significant change in the OPR fill rate per 100 000 county residents after Medicaid expansion in expansion counties vs nonexpansion counties (327.4; 95% CI, −202.5 to 857.4; *P* = .16) (Table 2). There was a significant increase in the rate of individuals filling OPRs paid for by Medicaid after the expansion in the expansion counties compared with nonexpansion counties of 374.0 per 100 000 population (95% CI, 258.3 to 489.7; *P* < .001). Contrary to hypothesis that there would be an offsetting decrease in cash payment, there were no significant changes in the rate of fills paid for by private insurance, cash, or Medicare. Similarly, there was no significant change in the number of days of the medication filled in Medicaid-expansion counties after expansion compared with non-Medicaid-expansion-counties.

**Table 2.** Difference-in-Differences Estimates for Opioid Pain Relievers and Buprenorphine With Naloxone^a^

Estimate	Overall Fills (All Payers)		Fill Rates per 100 000 Population by Payer			
	Rate per 100 000 Population^b^	No. of Days of Fill^c^	Medicaid	Cash	Private Insurance	Medicare
**Opioid Pain Relievers**						
Mean value in 2010	5298.3	88.1	859.5	1069.7	3941.4	410.6
Difference-in-differences estimate (95% CI)^d^	327.4 (−202.5 to 857.4)	2.9 (−1.0 to 6.7)	374.0 (258.3 to 489.7)	179.5 (−30.1 to 389.0)	−110.2 (−536.9 to 316.6)	−1.7 (−44.1 to 40.7)
*P* value	.16	.11	<.001	.08	.51	.92
Change relative to 2010, %	6.2	3.3	43.5	16.8	−2.8	−0.4
**Buprenorphine With Naloxone**						
Mean value in 2010	68.8	154.4	10.6	22.9	51.8	3.8
Difference-in-differences estimate (95% CI)^d^	8.7 (1.7 to 15.7)	−8.2 (−24.2 to 7.8)	9.3 (−1.6 to 20.1)	2.9 (−1.0 to 6.7)	−.3 (−2.1 to 1.5)	.01 (−1.2 to 1.3)
*P* value	.03	.23	.08	.11	.66	.98
Change relative to 2010, %	12.6	−5.3	87.7	12.7	−0.6	0.3

^a^ Analysis of IQVIA prescription claims data aggregated to county-years from California, Maryland, and Washington (Medicaid expansion counties) and Florida and Georgia (nonexpansion counties), N = 2082 county-years. County-years were weighted by the county population.

^b^ Represents the number of individuals in each county-year in our sample using the medication as a rate per 100 000 individuals in the county populations.

^c^ Mean number of days per person of filled medication.

^d^ Trend difference for individuals in Medicaid expansion counties pre- and post-expansion subtracted from the trend difference in nonexpansion counties in a model that also adjusts for baseline county characteristics and includes state and year fixed effects.

### Buprenorphine With Naloxone

**Figure 3.**
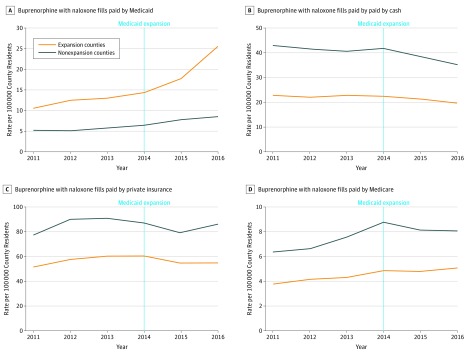
Trends in Rate of People Filling Prescriptions for Buprenorphine With Naloxone by Payer Analysis of IQVIA prescription claims data on prescriptions paid by Medicaid (A), cash (B), private insurance (C), and Medicare (D) aggregated to county-years from California, Maryland, and Washington (Medicaid expansion counties) and Florida and Georgia (nonexpansion counties), N = 2082 county-years. County-years are weighted by the county population.

For the 2010-2015 period, the rate per 100 000 residents of individuals filling buprenorphine with naloxone prescriptions increased in expansion counties from 68.8 to 77.1. In nonexpansion counties, the rate was 98.8 in 2010 and 99.2 in 2015 (Figure 1). The annual days of fills increased in expansion counties from 154.4 to 173.3 days and 135.2 to 172.1 days in nonexpansion counties (Figure 1). The rate of individuals with fills paid for by Medicaid per 100 000 increased from 10.6 to 25.4 in expansion counties and from 5.2 to 8.5 in nonexpansion counties (Figure 3). For other forms of payment, the rate of individuals paying for buprenorphine with naloxone with cash declined in both expansion and nonexpansion counties, but there were slight increases for private insurance and Medicare in both groups of counties.

In difference-in-differences analysis, there was a significant increase of 8.7 per 100 000 (95% CI, 1.7-15.7; *P* = .03) in the overall rate of individuals filling buprenorphine with naloxone prescriptions in expansion counties vs nonexpansion counties after Medicaid expansion (Table 2). Although this increase corresponds to a commensurate change in the rate of buprenorphine with naloxone fills per 100 000 county residents paid for by Medicaid (9.3; 95% CI, −1.6 to 20.1), this change was not statistically significant at conventional levels (*P* = .08). All changes by other payers were smaller and not statistically significant. There were no significant changes in the mean number of days of medication.

### Sensitivity Analyses

We did not find evidence that the parallel trends assumption was violated for any of our regression outcomes (eTable 1 in the Supplement). Analyses limited to individuals consistently observed in the data yielded results that were smaller, but in a consistent direction to, our main analyses (eTable 2 in the Supplement). Models that additionally tested for differences between counties with higher vs lower baseline uninsured rates did not find that a county’s baseline uninsured rate differentially influenced changes after Medicaid expansion (eTable 3 in the Supplement). Results held up in models that dropped each state 1 at a time, with consistent changes in magnitude and direction associated with fill rates overall and fills with Medicaid (eTable 4 in the Supplement). Finally, models that applied the inverse probability of treatment weights yielded results that were qualitatively similar to the main results but were less precisely estimated (eTable 5 and eTable 6 in the Supplement).

## Discussion

The ACA has expanded insurance to millions of Americans, yet little is known about whether the law changed use of OPRs or buprenorphine with naloxone. We used longitudinal, anonymized, all-payer claims from several states to examine this question. Consistent with other analyses,^25^ we found that the rate of OPRs from all sources of payment steadily declined overall during the study period, while rates of buprenorphine with naloxone fills steadily increased. There was no statistically significant change in the fill rate for OPRs in expansion counties. These results are important given how little is known about the result of Medicaid expansion on opioid utilization, as well as concerns that Medicaid expansion fueled the opioid epidemic.^26^

We found that the overall rate of buprenorphine with naloxone significantly increased in expansion counties relative to nonexpansion counties. Compared with the baseline rate of buprenorphine with naloxone, our analysis implies a roughly 13% increase in buprenorphine with naloxone use in the population. Prior studies have shown that the volume of buprenorphine claims paid by Medicaid specifically substantially increased after Medicaid expansion,^6,11^ but we believe that our study is the first to look across all payers. Our findings indicate that overall fills of buprenorphine with naloxone increased, and not merely those reimbursed by Medicaid, potentially indicating an increase overall in office-based treatment for opioid use disorder. Study findings are important to consider in light of the mixed evidence on whether expanding Medicaid increases opioid use disorder treatment.^11,27,28,29^

Our findings underscore the important role that Medicaid programs play in the opioid epidemic, especially in expansion states. State Medicaid programs have a variety of opportunities to encourage safer use of prescription opioids, including through the design of formularies and utilization management tools, such as prior authorization and quantity limits, that promote the use of evidence-based treatments for chronic noncancer pain.^30^ For example, Maryland and Washington Medicaid now have prior authorization requirements in place for patients seeking high-dosage or long-acting opioids.^31^ The effect of these efforts, as well as many other initiatives by a variety of stakeholders, might account for the reductions in OPR prescription volume that were observed during the study period, although further evaluation is needed to assess the degree to which these changes have reduced high-risk opioid prescriptions and nonmedical use as well as improved the management of pain.

Many Medicaid programs are also taking steps to increase access to buprenorphine for the treatment of opioid use disorder, which is a critical step to combat the current opioid crisis. Some states are focused on increasing the prescriber base by encouraging more physicians to obtain prescribing waivers and granting scope of practice to nurse practitioners and physician assistants, which is a change supported by recent federal regulations.^32^ The 21st Century Cures Act provides $1 billion to states over 2 years to undertake system reforms that could also increase buprenorphine access for patients, such as care integration models that link office-based prescribers to specialty treatment clinics. States are also revisiting their prior authorization rules to ensure that they do not hinder access to buprenorphine, although many states continue to maintain limitations on buprenorphine prescribing in Medicaid. Beyond increasing the number of patients in treatment, Medicaid programs are beginning to monitor quality of care, focusing on continuity of buprenorphine treatment and provision of appropriate psychosocial services and oversight of patients receiving buprenorphine.^33^

### Limitations

Our study has several limitations. Because we only included 5 states in our analysis, the findings may not represent the experience of other states. However, 26% of the US population resides in the 5 states that we included. Moreover, the 2 expansion states were not necessarily comparable to the nonexpansion states because of demographic and social differences that may have led them to follow different trends independent of ACA Medicaid expansion. Although we attempted to control for these variations through county demographic covariates and state-level fixed effects, there could be unobserved differences between the states that are associated with the outcomes that we measured. Some variables include a patient’s rate of physician office visits, locations of physicians who have buprenorphine waivers, and a patient’s utilization of other drug therapies for opioid use disorder, such as methadone, which is not captured in retail prescription data. Although we used the same study period as other recent studies of ACA Medicaid expansion,^34,35^ including more years prior to 2010 could improve our ability to establish the pre-expansion trends. Likewise, including years after 2015 could improve estimation of postexpansion change. Finally, we cannot rule out alternative explanations that may be driving prescription rates independent of the ACA. These could include state-specific changes adopted by payers to alter prior authorization for opioids, increased scrutiny of opioid prescribing, changed pain management practices, or expansions of buprenorphine prescriber capacity.

## Conclusions

Insurance expansions under the ACA occurred during a period of declining OPR fills and increasing buprenorphine with naloxone fills. In the 5 states that we studied, we found that Medicaid expansion was associated with more individuals overall filling buprenorphine with naloxone prescriptions. Although there was no significant overall change in OPR fills per 100 000 county residents, Medicaid expansion was associated with more individuals filling OPRs paid for by Medicaid specifically. The increasing role of Medicaid in covering populations seeking these treatments suggests the need for comprehensive efforts by state programs to track patients receiving OPRs, expand nonopioid options for pain care, screen for opioid use disorder, and link high-risk patients to evidence-based addiction treatments, such as treatment with buprenorphine with naloxone. The potential implications of these changes in prescription use on rates of addiction and overdose are an important area for future research.

## Notes

Supplement. **eTable 1.** Test of Pre-Treatment Parallel Trends **eTable 2.** Difference-in-Differences Estimates for Constant Sample **eTable 3.** Triple Differences Models That Include Interaction with Baseline County Uninsured Rate **eTable 4.** Difference-in-Differences Models Subtracting Each State **eTable 5.** Difference-in-Differences Estimates Using Inverse Probability of Treatment **eTable 6.** Balance of Covariates With Inverse Probability of Treatment Weighting (IPTW)

## References

[1] Clarke TC, Ward BW, Norris T, Schiller JS Early release of selected estimates based on data from the January-September 2016 National Health Interview Survey. https://www.cdc.gov/nchs/data/nhis/earlyrelease/earlyrelease201702.pdf. Accessed July 12, 2018.

[2] Hedegaard H, Warner M, Miniño AM Drug overdose deaths in the United States, 1999-2016. NCHS Data Brief. 2017;(294):-. 29319475

[3] A Majority Staff Report of the Committee on Homeland Security and Governmental Affairs United States Senate. Drugs for Dollars: How Medicaid Fuels the Opioid Epidemic. Washington, DC: US Senate; 2018.

[4] Goodman-Bacon A, Sandoe E Did Medicaid expansion cause the opioid epidemic? there’s little evidence that it did. https://www.healthaffairs.org/do/10.1377/hblog20170823.061640/full/. Published 2018. Accessed March 1, 2018.

[5] Broaddus M, Bailey P, Aron-Dine A Medicaid Expansion Dramatically Increased Coverage for People With Opioid-Use Disorders, Latest Data Show. Washington, DC: Center for Budget and Policy Priorities; 2018 https://www.cbpp.org/research/health/medicaid-expansion-dramatically-increased-coverage-for-people-with-opioid-use. Accessed March 1, 2018.

[6] Sharp A, Jones A, Sherwood J, Kutsa O, Honermann B, Millett G Impact of Medicaid Expansion on access to opioid analgesic medications and medication-assisted treatment. Am J Public Health. 2018;108(5):642-648. doi:10.2105/AJPH.2018.304338 29565661 PMC5888053

[7] Barry CL, Huskamp HA Moving beyond parity—mental health and addiction care under the ACA. N Engl J Med. 2011;365(11):973-975. doi:10.1056/NEJMp1108649 21848453 PMC3359059

[8] Saloner B, Karthikeyan S Changes in substance abuse treatment use among individuals with opioid use disorders in the United States, 2004-2013. JAMA. 2015;314(14):1515-1517. doi:10.1001/jama.2015.10345 26462001

[9] Chen KY, Chen L, Mao J Buprenorphine-naloxone therapy in pain management. Anesthesiology. 2014;120(5):1262-1274. doi:10.1097/ALN.0000000000000170 24509068 PMC3999180

[10] Roberts AW, Saloner B, Dusetzina SB Buprenorphine use and spending for opioid use disorder treatment: trends from 2003 to 2015. Psychiatr Serv. 2018;69(7):832-835. doi:10.1176/appi.ps.201700315 29734918 PMC6028283

[11] Wen H, Hockenberry JM, Borders TF, Druss BG Impact of Medicaid expansion on Medicaid-covered utilization of buprenorphine for opioid use disorder treatment. Med Care. 2017;55(4):336-341. doi:10.1097/MLR.0000000000000703 28296674

[12] Cope LC, Lynch V, Epstein M, Kenney G Medicaid Coverage of Effective Treatment for Opioid Use Disorder Trends in State Buprenorphine Prescriptions and Spending Since 2011. Washington, DC: The Urban Institute; 2017.

[13] Saloner B, Daubresse M, Caleb Alexander G Patterns of buprenorphine-naloxone treatment for opioid use disorder in a multistate population. Med Care. 2017;55(7):669-676. doi:10.1097/MLR.0000000000000727 28410339 PMC6528471

[14] Chang H-Y, Lyapustina T, Rutkow L, Impact of prescription drug monitoring programs and pill mill laws on high-risk opioid prescribers: a comparative interrupted time series analysis. Drug Alcohol Depend. 2016;165:1-8. doi:10.1016/j.drugalcdep.2016.04.033 27264166 PMC4985620

[15] Centers for Disease and Control and Prevention Analyzing prescription data and morphine milligram equivalents (MME). September 2017 https://www.cdc.gov/drugoverdose/resources/data.html. Accessed May 16, 2018.

[16] Golberstein E, Gonzales G, Sommers BD California’s early ACA expansion increased coverage and reduced out-of-pocket spending for the state’s low-income population. Health Aff (Millwood). 2015;34(10):1688-1694. doi:10.1377/hlthaff.2015.0290 26438745 PMC4769999

[17] US Census Bureau American Community Survey (ACS), 2018 https://www.census.gov/programs-surveys/acs/. Accessed July 3, 2018.

[18] US Department of Health and Human Services HRSA Data Warehouse; Area Health Resource File: 2018 https://datawarehouse.hrsa.gov/topics/ahrf.aspx. Accessed July 3, 2018.

[19] Centers for Disease Control and Prevention Drug Overdose Death Data. 2018 https://www.cdc.gov/drugoverdose/data/statedeaths.html. Accessed July 3, 2018.

[20] Karaca-Mandic P, Norton EC, Dowd B Interaction terms in nonlinear models. Health Serv Res. 2012;47(1, pt 1):255-274. doi:10.1111/j.1475-6773.2011.01314.x 22091735 PMC3447245

[21] Williams RL A note on robust variance estimation for cluster-correlated data. Biometrics. 2000;56(2):645-646. doi:10.1111/j.0006-341X.2000.00645.x 10877330

[22] Sawilowsky SS New effect size rules of thumb. J Mod Appl Stat Methods. 2009;8(2):597-599. doi:10.22237/jmasm/1257035100

[23] Courtemanche C, Marton J, Ukert B, Yelowitz A, Zapata D Early impacts of the Affordable Care Act on health insurance coverage in Medicaid expansion and non-expansion states. J Policy Anal Manage. 2017;36(1):178-210. doi:10.1002/pam.21961 27992151

[24] Dugoff EH, Schuler M, Stuart EA Generalizing observational study results: applying propensity score methods to complex surveys. Health Serv Res. 2014;49(1):284-303. doi:10.1111/1475-6773.12090 23855598 PMC3894255

[25] Centers for Disease and Control and Prevention Annual surveillance report of drug-related risks and outcomes. https://stacks.cdc.gov/view/cdc/47832. Published 2017. Accessed March 1, 2018.

[26] Finley A Does Medicaid spur opioid abuse? *Wall Street Journal* https://www.wsj.com/articles/does-medicaid-spur-opioid-abuse-1506289279. Published September 24, 2017. Accessed March 1, 2018.

[27] Baicker K, Allen HL, Wright BJ, Finkelstein AN The effect of Medicaid on medication use among poor adults: evidence from Oregon. Health Aff (Millwood). 2017;36(12):2110-2114. doi:10.1377/hlthaff.2017.0925 29200347 PMC5739033

[28] Maclean JC, Saloner B The Effect of Public Insurance Expansions on Substance Use Disorder Treatment: Evidence From the Affordable Care Act. Cambridge, MA: National Bureau of Economic Research; 2017. doi:10.3386/w23342 PMC7071834 30882195

[29] Feder KA, Mojtabai R, Krawczyk N, Trends in insurance coverage and treatment among persons with opioid use disorders following the Affordable Care Act. Drug Alcohol Depend. 2017;179:271-274. doi:10.1016/j.drugalcdep.2017.07.015 28823834 PMC5612778

[30] Wen H, Schackman BR, Aden B, Bao Y States with prescription drug monitoring mandates saw a reduction in opioids prescribed to Medicaid enrollees. Health Aff (Millwood). 2017;36(4):733-741. doi:10.1377/hlthaff.2016.1141 28373340 PMC5625882

[31] National Conference on State Legislatures. *Prescribing Policies: States Confront Opioid Overdose Epidemic* Washington, DC: National Conference on State Legislatures; 2017.

[32] Fornili KS, Fogger SA Nurse practitioner prescriptive authority for buprenorphine: from DATA 2000 to CARA 2016. J Addict Nurs. 2017;28(1):43-48. doi:10.1097/JAN.0000000000000160 28252511

[33] Medicaid Payment Advisory Committee Medicaid and the Opioid Epidemic. Washington, DC: Medicaid Payment Advisory Committee; 2017.

[34] Griffith K, Evans L, Bor J The Affordable Care Act reduced socioeconomic disparities in health care access. Health Aff (Millwood). 2017;36(8):1503-1510. doi:10.1377/hlthaff.2017.0083 28747321 PMC8087201

[35] Miller S, Wherry LR Health and access to care during the first 2 years of the ACA Medicaid expansions. N Engl J Med. 2017;376(10):947-956. doi:10.1056/NEJMsa1612890 28273021

