# Supplementary material for: Changes in Buprenorphine-Naloxone and Opioid Pain Reliever Prescriptions After the Affordable Care Act Medicaid Expansion
Source: JAMA Netw Open. 2018 Aug 17;1(4):e181588. doi: 10.1001/jamanetworkopen.2018.1588 (PMC6324520; doi:10.1001/jamanetworkopen.2018.1588)
Supplement: Supplement. — eTable 1. Test of Pre-Treatment Parallel Trends eTable 2. Difference-in-Differences Estimates for Constant Sample eTable 3. Triple Differences Models That Include Interaction with Baseline County Uninsured Rate eTable 4. Difference-in-Differences Models Subtracting Each State eTable 5. Difference-in-Differences Estimates Using Inverse Probability of Treatment eTable 6. Balance of Covariates With Inverse Probability of Treatment Weighting (IPTW) [file jamanetwopen-1-e181588-s001.pdf]

Supplementary Online Content

Saloner B, Levin J, Chang H-Y, Jones C, Alexander GC. Changes in buprenorphine-naloxone and opioid pain reliever prescriptions after the Affordable Care Act Medicaid expansion. *JAMA Netw Open*. 2018;1(4):e181588. doi:10.1001/jamanetworkopen.2018.1588

eTable 1. Test of Pre-Treatment Parallel Trends

eTable 2. Difference-in-Differences Estimates for Constant Sample

eTable 3. Triple Differences Models That Include Interaction with Baseline County Uninsured Rate

eTable 4. Difference-in-Differences Models Subtracting Each State

eTable 5. Difference-in-Differences Estimates Using Inverse Probability of Treatment

eTable 6. Balance of Covariates With Inverse Probability of Treatment Weighting (IPTW)

This supplementary material has been provided by the authors to give readers additional information about their work.

© 2018 Saloner B et al. *JAMA Network Open.*

© 2018 Saloner B et al. *JAMA Network Open.*

eTable 1. Test of Pre-Treatment Parallel Trends

	Overall Fills (All-Payers)		Fill Rates Per 100,000 Population by Payer			
	**Rate Per 100,000 Population**	**Number of days of fill**	**Medicaid**	**Cash**	**Private insurance**	**Medicare**
**Opioid Pain Relievers**	103.6	0.81	16.5	60.0	78.7	-16.6
(95% CI) [P-value]	(-24.2 to 231.4) [0.087]	(-2.38 to 4.00) [0.520]	(-35.4, 68.4) [0.427]	(-13.1 to 133.1) [0.085]	(-18.1 to 175.5) [0.087]	(-39.5, 6.4) [0.115]
**Buprenorphine-Naloxone**	0.12	-6.29	0.57	-0.15	-1.46	-0.60
(95% CI) [P-value]	(-5.15 to 5.39) [0.954]	(-13.2 to 0.64) [0.065]	(-2.32 to 3.47) [0.613]	(-1.62 to 1.31) [0.784]	(-5.94 to 3.02) [0.416]	(-1.50 to 0.30) [0.137]

Notes: The test of pre-treatment parallel trends reports on a coefficient representing the interaction between "expansion county" and year in all years prior to the expansion of Medicaid in a model that also adjusts for baseline county characteristics and includes state and year fixed effects.

© 2018 Saloner B et al. *JAMA Network Open.*

eTable 2. Difference-in-Differences Estimates for Constant Sample

	Overall Fills (All-Payers)		Fill Rates Per 100,000 Population by Payer			
**Opioid Pain Relievers**	**Rate Per 100,000 Population**	**Number of days of fill**	**Medicaid**	**Cash**	**Private insurance**	**Medicare**
Mean Value in 2010	2723.5	105.1	515.3	465.4	2411.7	305.9
Difference-in-Differences Estimate (95% CI) [P-value]	152.9	3.7	145.0	78.3	88.5	-16.2
	(-110.5 to 416.4) [0.182]	(-2.6 to 9.9) [0.178]	(34.6 to 255.3) [0.022]	(8.0 to 148.6) [0.037]	(-204.6 to 381.7) [0.449]	(-87.7 to 55.3) [0.563]
Change Relative to 2010	5.6%	3.5%	28.1%	16.8%	3.7%	-5.3%
**Buprenorphine-Naloxone**						
Mean Value in 2010	37.0	170.2	5.4	10.0	26.3	2.1
Difference-in-Differences Estimate (95% CI) [P-value]	4.0	-13.5	3.7	-0.6	-2.2	-0.4
	(-3.5 to 11.4) [0.213]	(-27.1 to 0.08) [0.051]	(-2.0 to 9.4) [0.149]	(-2.3 to 1.1) [0.390]	(-5.7 to 1.3) [0.154]	(-1.8 to 1.1) [0.515]
Change Relative to 2010	10.8%	-7.9%	68.5%	-6.0%	-8.4%	-19.0%

Notes: These estimates report on the same models as Tables 2 in the text, but restrict to a sample of individuals in the study counties who have claims appearing in all years of the data (a constant sample).

© 2018 Saloner B et al. *JAMA Network Open.*

eTable 3. Triple Differences Models That Include Interaction with Baseline County Uninsured Rate

	Overall Fills (All-Payers)		Fill Rates Per 100,000 Population by Payer			
**Opioid Pain Relievers**	**Rate Per 100,000 Population**	**Number of days of fill**	**Medicaid**	**Cash**	**Private insurance**	**Medicare**
Mean Value in 2010	5298.3	88.1	859.5	1069.7	3941.4	410.6
Difference-in-Difference-in-Differences Estimate (95% CI) [P-value]	22.2	4.6	-51.1	-54.1	-115.1	-58.0
	(-861.1 to 905.4) [0.948]	(-3.6 to 12.8) [0.196]	(-173.6 to 71.4) [0.311]	(-197.0 to 88.7) [0.352]	(-721.4 to 491.2) [0.626]	(-195.6 to 79.6) [0.307]
Change Relative to 2010	0.4%	5.2%	-5.9%	-5.1%	-2.9%	-14.1%
**Buprenorphine-Naloxone**						
Mean Value in 2010	68.8	154.4	10.6	22.9	51.8	3.8
Difference-in-difference-in-differences Estimate (95% CI) [P-value]	-1.8	-4.6	-6.9	-4.4	-2.2	-0.05
	(-24.5 to 20.8) [0.834]	(-15.2 to 6.0) [0.296]	(-17.3 to 3.5) [0.138]	(-8.3 to -0.48) [0.036]	(-18.6 to 14.2) [0.729]	(-0.78 to 0.69) [0.874]
Change Relative to 2010	-2.6%	-3.0%	-65.1%	-19.2%	-4.2%	-1.3%

Notes: These estimates report on the same models as Tables 2 in the text, but include an additional interaction between being observed in a county with higher than median uninsured rate in the baseline period (testing for differential effects in the subset of counties that began with higher uninsured rates).

© 2018 Saloner B et al. *JAMA Network Open.*

eTable 4. Difference-in-Differences Models Subtracting Each State

	Overall Fills (All-Payers)		Fill Rates Per 100,000 Population by Payer			
**Opioid Pain Relievers**	**Rate Per 100,000 Population**	**Number of days of fill**	**Medicaid**	**Cash**	**Private insurance**	**Medicare**
Without CA, DD Estimate (95% CI) [p-value]	-127.2	0.9	313.3	57.6	-426.5	-17.3
	(-955.5 to 701.1) [0.659]	(-3.2 to 4.9) [0.547]	(25.9 to 600.8) [0.040]	(-335.6 to 450.8) [0.673]	(-1251.2 to 398.3) [0.198]	(-171.7 to 137.1) [0.745]
Without WA	493.2	3.1	363.3	241.3	33.3	11.5
	(254.9 to 731.6) [0.007]	(0.9 to 5.3) [0.021]	(187.6 to 539.0) [0.007]	(95.4 to 387.2) [0.013]	(-136.4 to 203.0) [0.576]	(-26.9 to 49.9) [0.411]
Without MD	350.3	2.5	404.5	174.1	-104.2	-7.0
	(-396.0 to 1096.5) [0.232]	(-2.09 to 7.0) [0.184]	(341.0 to 467.9) [<0.001]	(-123.4 to 471.7) [0.159]	(-739.0 to 530.6) [0.637]	(-67.9 to 53.9) [0.740]
Without GA	289.1	2.4	363.9	206.2	-134.0	6.6
	(-272.5 to 850.7) [0.200]	(-0.9 to 5.7) [0.105]	(211.1 to 516.8) [0.005]	(-14.2 to 426.5) [0.059]	(-584.4 to 316.5) [0.414]	(-33.4 to 46.7) [0.634]
Without FL	301.2	0.9	376.2	105.5	-137.3	-16.5
	(-174.3 to 776.8) [0.137]	(-3.5 to 5.4) [0.549]	(260.2 to 492.2) [0.002]	(-70.9 to 281.9) [0.153]	(-571.3 to 296.7) [0.388]	(-66.5 to 33.4) [0.369]
**Buprenorphine-Naloxone**						
Without CA	9.9	-2.1	16.0	-0.4	-1.5	0.3
	(3.0 to 16.8) [0.02]	(-15.5 to 11.3) [0.654]	(10.7 to 21.4) [0.002]	(-9.2 to 8.3) [0.885]	(-11.4 to 8.4) [0.661]	(-1.2 to 1.7) [0.602]
Without WA	9.4	-10.0	7.8	3.1	-0.4	0.09
	(-0.86 to 19.7) [0.062]	(-29.1 to 9.0) [0.192]	(-5.3 to 20.9) [0.154]	(-2.4 to 8.6) [0.173]	(-3.6 to 2.9) [0.724]	(-1.7 to 1.9) [0.877]
Without MD	6.2	-9.5	6.5	3.8	0.06	-0.3
	(0.64 to 11.7) [0.038]	(-28.4 to 9.4) [0.207]	(-5.1 to 18.1) [0.171]	(2.0 to 5.6) [0.007]	(-2.8 to 2.9) [0.948]	(-1.7 to 1.1) [0.538]
Without GA	9.2	-12.6	8.7	2.4	-0.6	-0.3
	(4.0 to 14.5) [0.011]	(-22.6 to -2.6) [0.028]	(-2.4 to 19.7) [0.089]	(-3.2 to 7.9) [0.265]	(-3.7 to 2.6) [0.613]	(-1.4 to 0.9) [0.518]

© 2018 Saloner B et al. *JAMA Network Open.*

Without FL	6.4	1.1	9.6	3.7	-0.2	0.5
	(-1.1 to 13.9) [0.073]	(-5.3 to 7.6) [0.620]	(-1.1 to 20.2) [0.064]	(1.2 to 6.2) [0.019]	(-2.7 to 2.3) [0.808]	(0.13 to 0.82) [0.021]

Notes: These estimates report on the same models as Tables 2 in the text, but subtract each state one at a time to test the sensitivity of the estimates to that state's omission.

© 2018 Saloner B et al. *JAMA Network Open.*

eTable 5. Difference-in-Differences Estimates Using Inverse Probability of Treatment

	Overall Fills (All-Payers)		Fill Rates Per 100,000 Population by Payer			
**Opioid Pain Relievers**	**Rate Per 100,000 Population**	**Number of days of fill**	**Medicaid**	**Cash**	**Private insurance**	**Medicare**
Mean Value in 2010	5298.3	88.1	859.5	1069.7	3941.4	410.6
Difference-in-Difference-in-Differences Estimate (95% CI) [P-value]	550.5	1.4	210.2	224.5	207.4	25.7
	(-717.2 to 1818.3) [0.294]	(-3.2 to 5.9) [0.445]	(19.5 to 400.9) [0.038]	(-137.3 to 586.3) [0.160]	(-886.6 to 1301.4) [0.626]	(-168.4 to 219.8) [0.732]
Change Relative to 2010	10.4%	1.6%	24.5%	21.0%	5.3%	6.3%
**Buprenorphine-Naloxone**						
Mean Value in 2010	68.8	154.4	10.6	22.9	51.8	3.8
Difference-in-difference-in-differences Estimate (95% CI) [P-value]	11.1	-2.4	8.2	6.0	-0.3	0.7
	(-7.6 to 29.7) [0.172]	(-12.4 to 7.6) [0.545]	(-4.3 to 20.7) [0.143]	(0.14 to 11.8) [0.047]	(-8.3 to 7.8) [0.935]	(-1.4 to 2.8) [0.414]
Change Relative to 2010	16.1%	-1.6%	77.4%	26.2%	-0.6%	18.4%

Weighting (IPTW)

Note: These estimates report on the same models as Tables 2 in the text, but multiply the population weight by an additional weighting factor: the Inverse Probability of Treatment Weight (IPTW). These weights predict the likelihood of a county being in an expansion versus non-expansion state based on baseline covariates and then provide greater weight to non-expansion counties that are more similar to expansion counties.

© 2018 Saloner B et al. *JAMA Network Open.*

eTable 6. Balance of Covariates With Inverse Probability of Treatment Weighting (IPTW)

	County mean in expanding states in 2010	County mean in non-expanding states in 2010	Mean difference between groups
**Mean demographics of counties**			
Percent adults (age >18)	64.6%	62.6%	2.0% (-0.8%, 4.7%)
Percent non-Hispanic white	56.4%	50.6%	5.8% (-21.6%, 10.0%)
Percent non-Hispanic black	23.3%	19.6%	3.6% (-32.1%, 39.4%)
Percent non-Hispanic other	7.8%	5.1%	2.6% (-9.6%, 14.9%)
Percent Hispanic	17.0%	17.7%	-0.6% (-33.7%, 32.5%)
Percent female	50.6%	51.0%	-0.34% (-1.1%, 0.4%)
Percent rural	11.6%	13.1%	-1.5% (-13.7%, 10.7%)
Percent uninsured	20.6%	28.0%	-7.3% (-15.5%, 0.8%)
Median income	$49,866	$50,460	-$594 (-$18,568, $17381)
Primary care physician to population ratio	0.78	0.65	0.12 (-0.01, 0.3)
**County overdose death rate**			
<10 per 100,000 residents	16.7%	47.3%	-30.6% (-99.2%, 37.9%)
10-20 per 100,000 residents	57.9%	44.8%	13.0% (-43.3%, 69.3%)
>20 per 100,000 residents	25.5%	7.9%	17.6% (-29.1%, 64.3%)
Number of counties	121	226	

© 2018 Saloner B et al. *JAMA Network Open.*
